# Adaptation of cortical activity to sustained pressure stimulation on the fingertip

**DOI:** 10.1186/s12868-015-0207-x

**Published:** 2015-10-29

**Authors:** Yoon Gi Chung, Sang Woo Han, Hyung-Sik Kim, Soon-Cheol Chung, Jang-Yeon Park, Christian Wallraven, Sung-Phil Kim

**Affiliations:** Department of Brain and Cognitive Engineering, Korea University, Anam-5ga, Seongbuk-gu, Seoul, 136-713 Republic of Korea; Department of Biomedical Engineering, BK21+ Research Institute of Biomedical Engineering, College of Biomedical & Health Science, Konkuk University, Chungju, 380-701 Republic of Korea; Center for Neuroscience Imaging Research, Institute of Basic Science (IBS), Sungkyunkwan University, Suwon, 440-746 Republic of Korea; Department of Global Biomedical Engineering, Sungkyunkwan University, Suwon, 440-746 Republic of Korea; Department of Human and Systems Engineering, Ulsan National Institute of Science and Technology, UNIST-gil 50, Ulsan, 689-798 Republic of Korea

**Keywords:** Somatosensory cortex, Tactile adaptation, Pressure, Functional connectivity, fMRI

## Abstract

**Background:**

Tactile adaptation is a phenomenon of the sensory system that results in temporal desensitization after an exposure to sustained or repetitive tactile stimuli. Previous studies reported psychophysical and physiological adaptation where perceived intensity and mechanoreceptive afferent signals exponentially decreased during tactile adaptation. Along with these studies, we hypothesized that somatosensory cortical activity in the human brain also exponentially decreased during tactile adaptation. The present neuroimaging study specifically investigated temporal changes in the human cortical responses to sustained pressure stimuli mediated by slow-adapting type I afferents.

**Methods:**

We applied pressure stimulation for up to 15 s to the right index fingertip in 21 healthy participants and acquired functional magnetic resonance imaging (fMRI) data using a 3T MRI system. We analyzed cortical responses in terms of the degrees of cortical activation and inter-regional connectivity during sustained pressure stimulation.

**Results:**

Our results revealed that the degrees of activation in the contralateral primary and secondary somatosensory cortices exponentially decreased over time and that intra- and inter-hemispheric inter-regional functional connectivity over the regions associated with tactile perception also linearly decreased or increased over time, during pressure stimulation.

**Conclusion:**

These results indicate that cortical activity dynamically adapts to sustained pressure stimulation mediated by SA-I afferents, involving changes in the degrees of activation on the cortical regions for tactile perception as well as in inter-regional functional connectivity among them. We speculate that these adaptive cortical activity may represent an efficient cortical processing of tactile information.

## Background

Tactile adaptation temporally desensitizes the tactile sensory system after an exposure to sustained or repetitive tactile stimuli [1, 2]. It has been reported that perceived intensity during tactile adaptation exponentially decreases over time at the psychophysical level [3]. In addition, the responses of mechanoreceptive afferents also exponentially decrease during tactile adaptation at the afferent level [1, 2]. In line with these previous studies, the human brain activation would also exponentially decrease during tactile adaptation at the cortical level.

Tactile adaptation on the cortex has been explored in a number of neurophysiologic studies. An optical intrinsic imaging study in non-human primate has demonstrated changes in cortical activation with a decrease in the spatial extent of the responses of the primary somatosensory cortex (SI) during sustained vibrotactile stimulation (25 Hz) [4]. Recent human magnetoencephalography (MEG) (2 and 4 Hz) [5] and functional magnetic resonance imaging (fMRI) (18–26 Hz) [6] studies have reported that activities of the somatosensory and parietal cortical regions decreased over time during repetitive vibrotactile stimuli. However, to our knowledge, no human study has explored tactile adaptation at the cortical level to sustained pressure stimulation, which would be manifested in exponential decreases in cortical activation. SA-I afferents deliver information of such low-frequency mechanical stimuli (pressure) to the somatosensory cortical regions [7, 8] for encoding sustained indentation [9], tangential [10] and grip forces [11], sizes [12], curvatures [13–16], position [17], and torque direction [18] during object manipulation. Therefore, we hypothesize that the human brain would exhibit tactile adaptation at the cortical level along with the response profile of SA-I afferents to sustained pressure stimulation shown in the previous neurophysiologic studies [1, 2].

In the present study, we specifically focused on two aspects of cortical activities: temporal changes in cortical responses and inter-regional connectivity. First, we examined which regions (e.g., SI) exhibited distinct changes in cortical activation during tactile adaptation. Second, we used the functional connectivity analysis with blood oxygenation level-dependent (BOLD) signals from multiple cortical regions. The functional connectivity analysis method used in this study has been widely used to examine inter-regional interactions in task- or resting-states [19] as well as to characterize cortical networks in static mechanical stimulation [20]. Although there is no existing study addressing how functional connectivity on the cortex changes during tactile adaptation, we ventured a hypothesis that functional connectivity over certain cortical regions associated with tactile perception would also adapt to sustained pressure stimulation.

## Methods

### Participants

We recruited 21 healthy volunteers (ages of 24.19 ± 2.71, right-handed) with no history of neurological disorders or deficits in tactile sensation. All participants gave written informed consent for this study approved by the Korea University Institutional Review Board (KU-IRB-11-46-A-1).

### Pressure stimulation

**Fig. 1:**
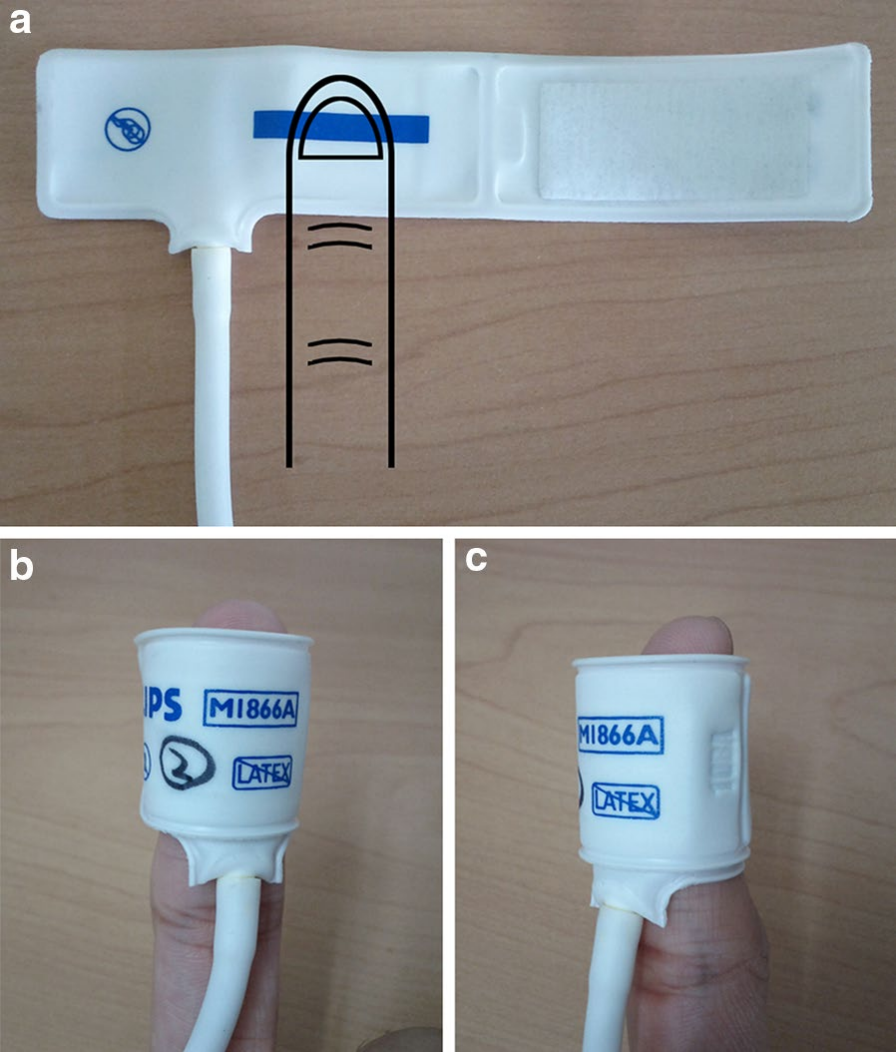
Pressure stimulation device. A right index fingertip was placed on a neonatal cuff (**a**) and was wrapped by the cuff (**b** *front view*, **c** *side view*) before MR scanning. The cuff was expanded by air insertion at the turn-on of the pump in a blood pressure monitor (not shown here) during MR scanning. In this way, a pressure stimulus of up to 58.6 kPa was applied to the whole ventral surface of the right index fingertip for 3, 9, or 15 s stimulation periods

We used a band-type MR compatible stimulation device developed by our group [21] that was able to apply pressure of up to 58.6 kPa (Fig. 1). A neonatal cuff (M1866A, Philips Healthcare, Best, The Netherlands) was directly connected to a rolling pump in a blood pressure monitor (BP3AG1, Microlife AG, Widnau, Switzerland) through an elastic air-tube with a length of 5 m and a diameter of 4 mm. The cuff wrapped an entire fingertip and was controlled by a pressure sensor for achieving uniform pressure. E-Prime 2.0 software (Psychology Software Tools, Inc., Sharpsburg, PA, USA) controlled the sensor to configure the length of stimulation. The cuff pressed the whole ventral surface of the fingertip by expanding through an air insertion when the pump was turned on. Detailed information about this stimulation device can be found in Kim et al. [21].

During each scanning session, the participants were instructed to lie on the MRI table comfortably and to close their eyes in order to prevent visual stimulation. During the entire scanning, the participants were requested to grab an emergency squeeze-ball weakly with their left hands. The squeeze-ball was not used for other purposes. The cuff wrapped the participants’ right index fingertip before scanning. It was confirmed that the cuff contacted the skin with no pressure so that participants verbally confirmed no perceived pressure before scanning and clearly perceived pressure during stimulation after each session. Each participant performed a series of three experiments, where each experiment corresponded to pressure stimulation with each of three different stimulation durations: 3, 9, or 15 s. An experiment consisted of four block-designed sessions where each session included four trials. A single trial was composed of a 21 s resting period followed by a 3, 9, or 15 s stimulation period. During each stimulation period, a single static indentation was applied continuously to the participant’s right index fingertip.

### Data acquisition

We performed MR scanning using a 3T MRI system (Magnetom TrioTim, Siemens Medical Systems, Erlangen, Germany) with a standard 32-channel head coil. T_1_-weighted anatomical images were acquired using a 3D magnetization-prepared gradient echo (MPRAGE) sequence with the imaging parameters of repetition time (TR) = 1900 ms, echo time (TE) = 2.48 ms, flip angle = 9°, field of view (FOV) = 200 mm, and voxel size = 0.8 × 0.8 × 1 mm^3^. T_2_*-weighted functional images were acquired using a gradient echo-planar imaging (EPI) sequence with the imaging parameters of TR = 3000 ms, TE = 30 ms, flip angle = 90°, FOV = 240 mm, slice thickness = 3 mm, and voxel size = 3 × 3 × 3 mm^3^.

### Statistical analysis on fMRI data

Preprocessing of the functional images was conducted using SPM8 (Wellcome Department of Imaging Neuroscience, UCL, London, UK). Preprocessing included the ordered sequence of data processing: slice-timing correction, realignment with the rigid-body transformation matrices, normalization to the Montreal Neurological Institute (MNI) template, and smoothing with an isotropic Gaussian kernel of 8 mm full-width-half-maximum (FWHM). The general linear model (GLM) in SPM8 was used to perform a statistical analysis of the BOLD signals with a canonical hemodynamic response function as well as its time and dispersion derivatives. A 128 s high-pass filter removed physiological artifacts in the BOLD signals. To observe time-varying cortical activation, stimulus durations of 9 or 15 s were divided into three (0–3, 3–6, and 6–9 s) or five stimulus segments (0–3, 3–6, 6–9, 9–12, and 12–15 s) with a time interval of 3 s for the GLM analysis. A random effects model was used to perform a group analysis for the inference of statistically significant cortical activations. Cluster-level *F* statistics [*p* < 0.05 with family-wise-error (FWE) correction, voxels for significant clusters (*k*) larger than 5] produced group-level statistical parametric maps (SPMs) representing significant voxel clusters. We obtained one, three, or five SPMs corresponding to individual stimulus segments in 3, 9, or 15 s pressure stimulation, respectively. The automated anatomical labeling toolbox [22] determined the anatomical cluster labels of activated regions in the SPMs.

We implemented independent one, three, and five design matrices for each condition of 3, 9 and 15 s simulation, respectively. In particular, we split the 15 s stimulus duration into five and the 9 s stimulus duration into three intervals in the GLM specification to perform statistical evaluation of cortical activation. For the 15 s stimulation, we constructed five separated box-car stimulus functions with five different onsets at 0, 3, 6, 9, and 12 s for each stimulus interval. Similarly, for the 9 s stimulation, we constructed three separated box–car stimulus functions with three different onsets at 0, 3, and 6 s for each stimulus interval. Each stimulus function was convolved with the canonical hemodynamic response function with time and dispersion derivatives to perform statistical inferences (*F*-test). Hence, cortical activation patterns from the first (0–3 s) to the last (6–9 or 12–15 s) stimulus intervals were analyzed using design matrices independently obtained by the separated stimulus functions.

The regressors of each stimulus interval (e.g., five regressors of 0–3, 3–6, 6–9, 9–12, and 12–15 s in case of the 15 s stimulation condition) may be highly correlated with each other if they belong to the same design matrix due to slow dynamics of the hemodynamic response function. However, in our study, we constructed design matrices for individual stimulus intervals independently with the separated box-car stimulus functions (as described above). Each design matrix contained only one type of regressor (e.g., five independent design matrices each having regressors of 0–3, 3–6, 6–9, 9–12, and 12–15 s, respectively). Our GLM analysis for each stimulation event was then performed using each of these separated design matrices. Consequently, we could avoid correlations between regressors in the GLM.

### Regions of interests (ROIs)

We used the Anatomy toolbox (version 1.8) [23] to generate anatomical masks in nine ROIs relevant to tactile information processing. They included the contralateral Brodmann area (cBA) 3, cBA1, and cBA2 at SI [24]; cBA40) and iBA40 at SII [25]; cBA5 and iBA5 at PPC neighboring on SI [24, 26, 27] known as the somatosensory association cortex [28]; and cBA13 and iBA13 at the insula neighboring on SII [28]. The number of suprathreshold voxels in these ROIs (group analysis with 21 subjects, *F*-test, *p*_(FWE)_ < 0.05, *k* > 5) was used in the following generalized linear model analysis.

### Generalized linear model analysis

We built a generalized linear model [29] to depict how the number of suprathreshold voxels in the ROIs decreased with time (the degree of cortical activation). To model a relationship between the number of suprathreshold voxels (*y*) and the stimulus duration (*t* = 3, 6, 9, 12, and 15 s), we used a log-linear function given as:

1 $$ y = ce^{ - (t/\tau )} = ce^{{(\alpha_{0} + \alpha_{1} t)}} $$

2 $$ \ln (y) = \alpha_{0} + \alpha_{1} t $$

where *c* and *α*_*0*_ are constants. We implemented a log-linear model instead of a linear model because we assumed that cortical adaptation would represent exponential characteristics likewise psychophysical [3] and afferent adaptation [1, 2]. The inverse and negative of the coefficient *α*_*1*_ was equivalent to a time constant (*τ*), representing a cortical adaptation rate. The statistical significance of the generalized linear model for each ROI was evaluated using the *F*-test (*p* < 0.05, uncorrected). Additionally, we compared the goodness of fit of the results from the log-linear (*r*^2^) and simple linear ($r_{\text{linear}}^{ 2}$) functions.

### Functional connectivity

We used the Conn toolbox (http://www.nitrc.org/projects/conn) [19] to investigate functional connectivity during the pressure stimulation. Realignment parameters selected as the first-level covariates were regressed out from the preprocessed functional images. Confounds were removed based on the aCompCor strategy [30], including effects in white matter, cerebrospinal fluid (CSF), realignment parameters and their first temporal derivatives, and main session effects and their first temporal derivatives. BOLD time series were band-pass filtered (0.0083 Hz < *f* < Inf) for removal of low-frequency drifts [31]. In the first-level analysis, we assessed functional connectivity among the nine ROIs defined above. ROI–ROI functional connectivity was measured by calculating an inter-regional bivariate correlation coefficient (*r*) between two BOLD signals and averaged over all the voxels in source and target ROIs. The averaged correlation coefficient value was then adjusted to a Fisher-transformed correlation coefficient, i.e., atanh(*r*), with a false-discovery rate (FDR) corrected threshold of *p* < 0.05 (one-sided, positive). After finding the correlation coefficients for all ROI–ROI pairs (36 pairs in total with no directionality and no self-connection), we performed a linear regression analysis between the correlation coefficients (*z*) and the stimulus duration (*t* = 3, 6, 9, 12, and 15 *s*) in each pair as follows:

3 $$ z = \beta_{0} + \beta_{1} t $$

The statistical significance of the linear model for each ROI–ROI pair was evaluated using the *F*-test (*p* < 0.05).

## Results

### Cortical activation

**Fig. 2:**
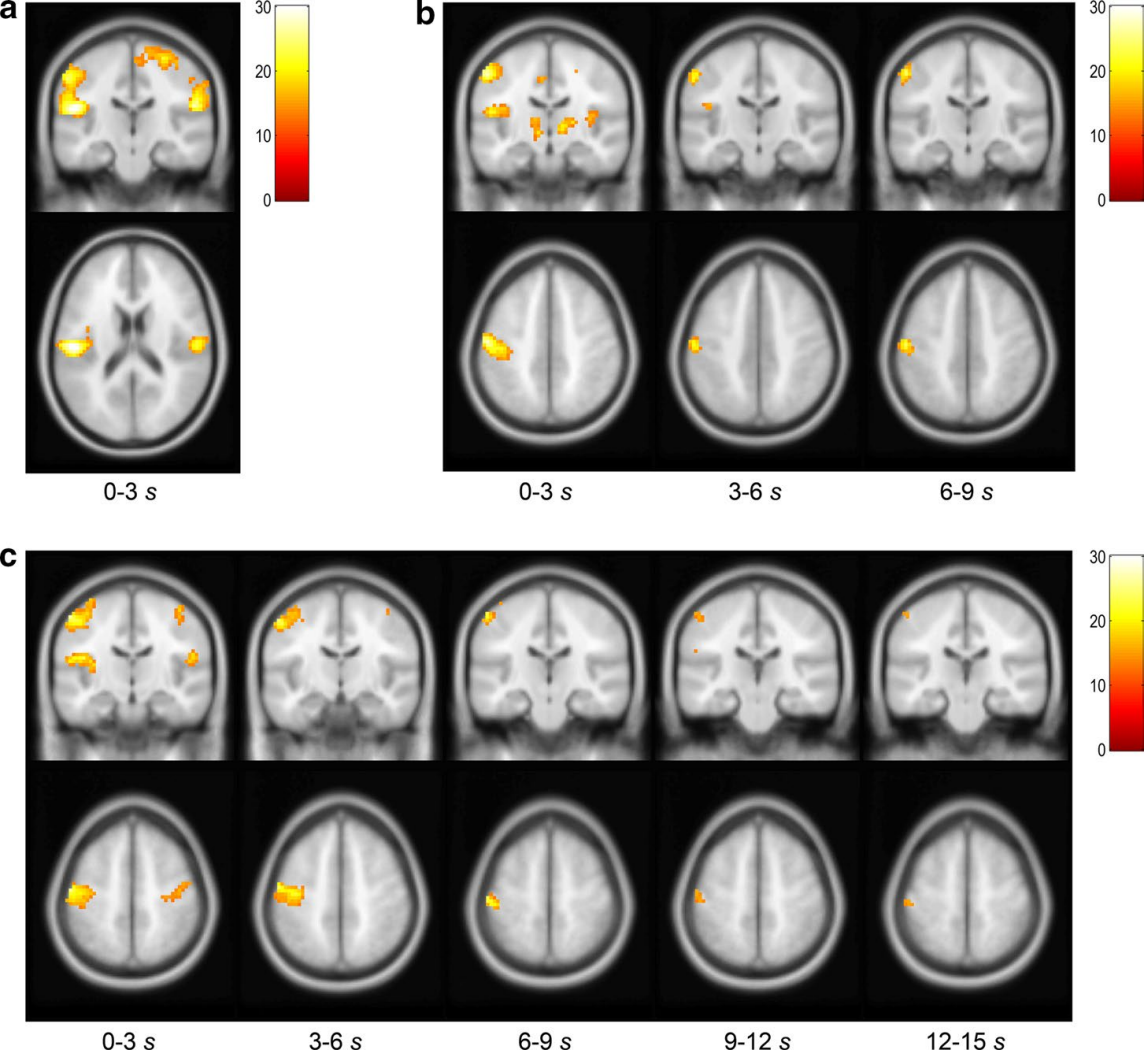
Cortical activation during pressure stimulation. Cortical activation patterns were investigated during 3 (**a**), 9 (**b**), and 15 s (**c**) pressure stimulation (group analysis with 21 participants, *p*_(FWE)_ < 0.05, *k* > 5, bar: *F*-statistics). Significantly activated clusters at the contralateral postcentral gyrus and bilateral rolandic operculum were consistently appeared during 3 s stimulation as well as during the first stimulus segment (0–3 s) of 9 and 15 s stimulation (cortical activation for tactile perception). During 15 s pressure stimulation, activated regions became localized in the contralateral postcentral gyrus (**c**)

The GLM group analysis revealed the clusters of significant cortical activation during 3, 9, and 15 s pressure stimulation (Fig. 2). The coordinates and statistical information of these clusters are summarized in Tables 1, 2, and 3 for 3, 9, and 15 s pressure stimulation, respectively. In particular, the clusters at the contralateral postcentral gyrus and bilateral rolandic operculum were consistently activated during 3 s stimulation as well as during the first stimulus segment (0–3 s) of 9 and 15 s stimulation, which confirmed cortical activation engaged in tactile perception [6, 32–38]. During 15 s pressure stimulation, activated regions became localized in the contralateral postcentral gyrus, without significant activation in the bilateral rolandic operculum.

**Table 1:** Activated regions during 3 s pressure stimulation (group analysis with 21 subjects, *F*-test, *p*_(FWE)_ < 0.05, *k* > 5)

Pressure		Anatomical labels		MNI coordinates (mm)			Voxels	*p*_(FWE)_	*F*	*Z*
				X	Y	Z				
3 s	0–3 s	Insula	R	42	3	9	116	0.0000	38.41	7.43
				39	−3	−3		0.0000	23.81	6.20
		Postcentral gyrus	L	−51	−21	18	527	0.0000	32.57	7.00
				−39	−3	9		0.0000	30.26	6.81
				−57	−21	45		0.0000	23.71	6.19
		Rolandic operculum	R	54	−21	21	220	0.0000	26.79	6.50
				60	−18	33		0.0004	19.38	5.68
				51	−21	45		0.0167	14.60	4.99
		Precentral gyrus	R	30	−24	60	207	0.0000	23.12	6.13
				24	−15	72		0.0001	20.81	5.86
				12	−27	72		0.0008	18.51	5.57
		Median cingulate	L	−6	9	36	6	0.0030	16.85	5.34
		Insula	R	33	27	3	22	0.0036	16.61	5.30
		Insula	L	−30	21	6	8	0.0080	15.61	5.15
		Paracentral lobule	L	−9	−27	66	11	0.0178	14.52	4.98

**Table 2:** Activated regions during 9 s pressure stimulation (group analysis with 21 subjects, *F*-test, *p*_(FWE)_ < 0.05, *k* > 5)

Pressure		Anatomical labels		MNI coordinates (mm)			Voxels	*p*_(FWE)_	*F*	*Z*
				x	y	z				
9 s	0–3 s	Postcentral gyrus	L	−54	−15	48	224	0.0000	26.98	6.52
				−48	−21	45		0.0000	26.57	6.48
		Rolandic operculum	L	−48	−21	18	195	0.0000	24.32	6.25
				−48	−3	6		0.0011	18.17	5.52
		Cerebellum	R	18	−51	−27	179	0.0000	22.96	6.11
				33	−63	−18		0.0014	17.86	5.48
				18	−57	−15		0.0149	14.76	5.02
		Thalamus	R	12	−12	0	82	0.0001	21.27	5.91
				21	−15	6		0.0008	18.61	5.58
				9	−21	−12		0.0171	14.57	4.99
		Median cingulate	L	−9	−18	45	18	0.0005	19.08	5.64
				−12	−27	45		0.0327	13.69	4.84
		Thalamus	L	−9	−15	−3	72	0.0008	18.62	5.58
				−18	−21	9		0.0008	18.55	5.57
				−9	−12	9		0.0011	18.08	5.51
		Cerebellum	L	−18	−39	−27	14	0.0013	17.93	5.49
		Insula	R	36	−15	9	24	0.0049	16.25	5.25
		Lingual gyrus	L	−21	−102	−15	14	0.0051	16.19	5.24
		Precentral gyrus	R	27	−12	51	6	0.0064	15.91	5.20
		Anterior cingulate	R	9	39	12	6	0.0092	15.42	5.12
		Rolandic operculum	R	48	−21	18	22	0.0105	15.24	5.09
				51	-30	12		0.0132	14.92	5.04
	4–6 s	Postcentral gyrus	L	−57	−18	45	51	0.0001	21.09	5.89
		Caudate	L	−27	−12	27	27	0.0007	18.74	5.60
				−21	−12	12		0.0111	15.25	5.10
		Cerebellum	R	27	−66	−21	44	0.0015	17.68	5.46
				24	−57	−21		0.0046	16.31	5.26
		Inferior frontal gyrus, opercular	R	30	−3	27	27	0.0017	17.54	5.44
		Inferior frontal gyrus, triangular	R	24	18	24	36	0.0018	17.46	5.43
				27	27	21		0.0053	16.14	5.23
				24	27	9		0.0142	14.90	5.04
		Anterior cingulate	R	18	36	21	7	0.0028	16.94	5.35
		Cerebellum	L	−15	−90	−24	85	0.0031	16.83	5.33
				−27	−84	−24		0.0038	16.56	5.30
				−6	−87	−24		0.0101	15.35	5.11
		Middle frontal gyrus	L	−21	18	33	6	0.0035	16.65	5.31
		Caudate	R	21	9	27	6	0.0047	16.28	5.25
		Rolandic operculum	L	−42	−18	21	11	0.0070	15.80	5.18
		Thalamus	R	9	−6	6	9	0.0113	15.22	5.09
	7–9 s	Postcentral gyrus	L	−54	−18	48	61	0.0000	23.48	6.16
		Cerebellum	R	27	−66	−24	46	0.0001	20.96	5.88
		Thalamus	R	12	−6	6	27	0.0003	19.78	5.73
		Cerebellum	L	−18	−39	−27	6	0.0036	16.62	5.30
		Lingual gyrus	L	−36	−84	−18	24	0.0037	16.58	5.30
		Middle frontal gyrus	R	27	24	18	7	0.0046	16.33	5.26
		Anterior cingulate	R	12	36	12	17	0.0070	15.80	5.18
		Middle frontal gyrus	R	24	48	30	8	0.0077	15.68	5.16
		Paracentral lobule	L	−15	−24	75	6	0.0115	15.19	5.09

**Table 3:** Activated regions during 15 s pressure stimulation (group analysis with 21 subjects, *F*-test, *p*_(FWE)_ < 0.05, *k* > 5)

Pressure		Anatomical labels		MNI coordinates (mm)			Voxels	*p*_(FWE)_	*F*	*Z*
				x	y	z				
15 s	0–3 s	Rolandic operculum	L	−48	−21	18	211	0.0000	28.14	6.62
				−45	−6	12		0.0007	18.65	5.59
				−33	−15	12		0.0009	18.46	5.56
		Postcentral gyrus	L	−54	−15	54	278	0.0000	26.06	6.43
				−36	−15	69		0.0025	17.08	5.37
		Rolandic operculum	R	54	−18	18	58	0.0004	19.53	5.70
		Median cingulate	L	−12	−21	48	9	0.0009	18.36	5.55
		Precentral gyrus	R	42	−15	60	102	0.0016	17.62	5.45
				51	−9	48		0.0088	15.53	5.14
				45	−9	54		0.0089	15.51	5.14
		Cerebellum	R	21	−48	−27	22	0.0040	16.50	5.29
		Postcentral gyrus	R	39	−30	69	12	0.0078	15.67	5.16
	4–6 s	Postcentral gyrus	L	−54	−12	51	281	0.0000	23.74	6.19
				−39	−18	51		0.0001	21.85	5.98
		Postcentral gyrus	R	36	−33	69	8	0.0024	17.15	5.38
		Precentral gyrus	R	42	−15	60	21	0.0057	16.06	5.22
				36	−21	60		0.0089	15.50	5.13
		Rolandic operculum	L	−45	−18	18	6	0.0161	14.68	5.00
	7–9 s	Postcentral gyrus	L	−54	−21	57	76	0.0001	22.29	6.03
	10–12 s	Rolandic operculum	L	−45	−18	18	27	0.0029	16.89	5.34
				−57	−21	18		0.0099	15.34	5.11
		Postcentral gyrus	L	−51	−24	54	29	0.0084	15.57	5.15
				−54	−15	54		0.0089	15.49	5.13
				−51	−18	42		0.0196	14.41	4.96
	13–15 s	Postcentral gyrus	L	−51	−24	57	8	0.0109	15.27	5.10

### Tactile adaptation on the cortex

**Fig. 3:**
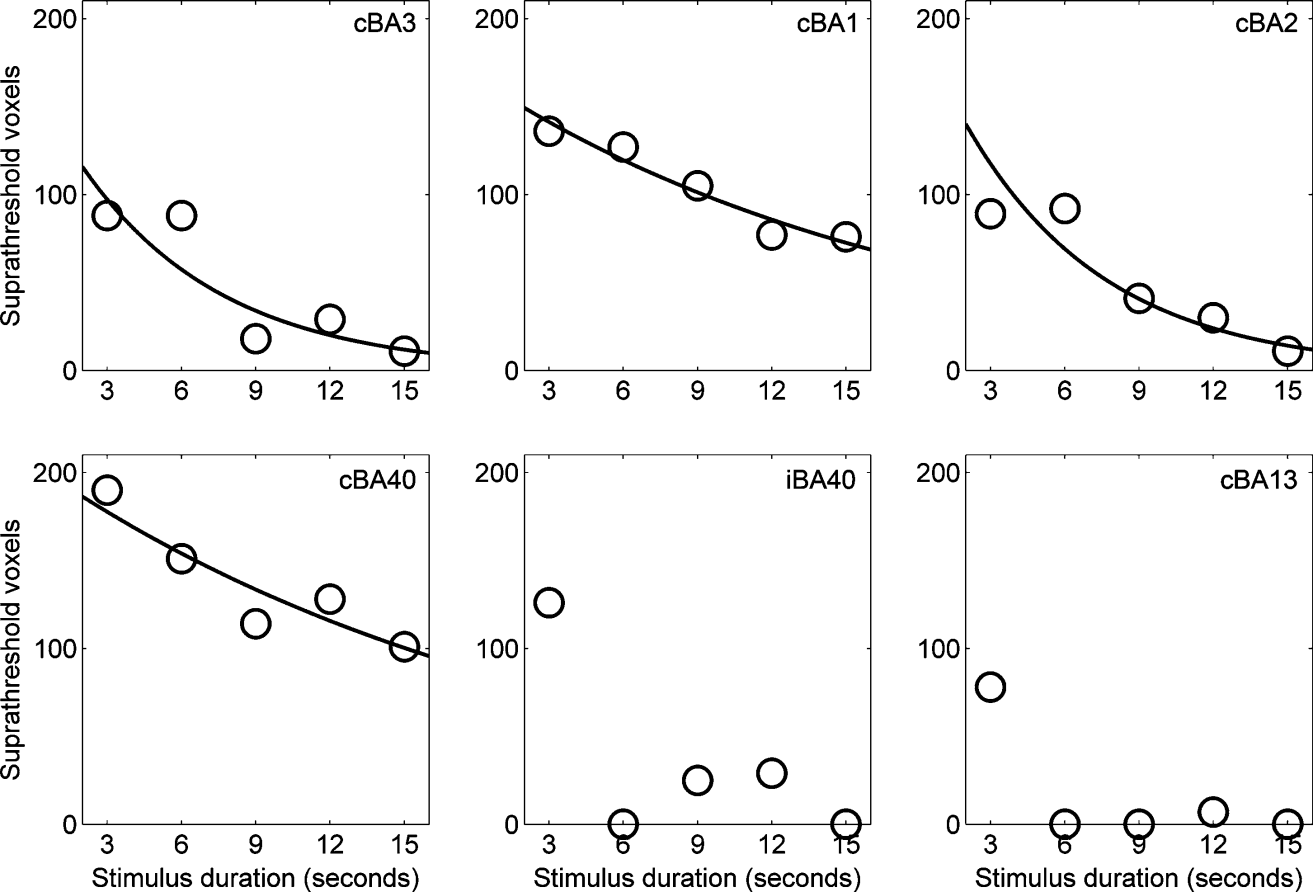
Exponential decreases in cortical activation during pressure stimulation. The number of suprathreshold voxels (*y*-axis) in cBA3 (*p* < 0.05, *τ* = 5.69 *s*), cBA1 (*p* < 0.01, *τ* = 18.03 *s*), cBA2 (*p* < 0.05, *τ* = 5.66 *s*), and cBA40 (*p* < 0.05, *τ* = 20.99 *s*) significantly decreased during 15 s pressure stimulation (generalized linear model analysis with a log-linear function). However, no significant change was shown in iBA40 and cBA13. iBA13, cBA5, and iBA5 also had no significant change (not shown here)

The generalized linear model analysis revealed significant exponential decreases in the number of suprathreshold voxels over particular cortical regions during 15 s pressure stimulation (Fig. 3). Specifically, the number of suprathreshold voxels in the following ROIs exponentially decreased over time: cBA3 (*r*^2^ = 0.79, *τ* = 5.69 *s*, *p* < 0.05, $r_{\text{linear}}^{ 2}$ = 0.78), cBA1 (*r*^2^ = 0.93, *τ* = 18.03 *s*, *p* < 0.01, $r_{\text{linear}}^{ 2}$ = 0.94), cBA2 (*r*^2^ = 0.91, *τ* = 5.66 *s*, *p* < 0.05, $r_{\text{linear}}^{ 2}$ = 0.90), and cBA40 (*r*^2^ = 0.84, *τ* = 20.99 *s*, *p* < 0.05, $r_{\text{linear}}^{ 2}$ = 0.82). The resulting time constant indicated that adaptation occurred faster in cBA2 and cBA3 than in cBA1 and cBA40. The number of suprathreshold voxels did not show any significant decreases over time in other ROIs, including the ipsilateral Brodmann area (iBA) 40, cBA13, and iBA13. No suprathreshold voxel was found in cBA5 and iBA5.

### Functional connectivity during tactile adaptation

**Fig. 4:**
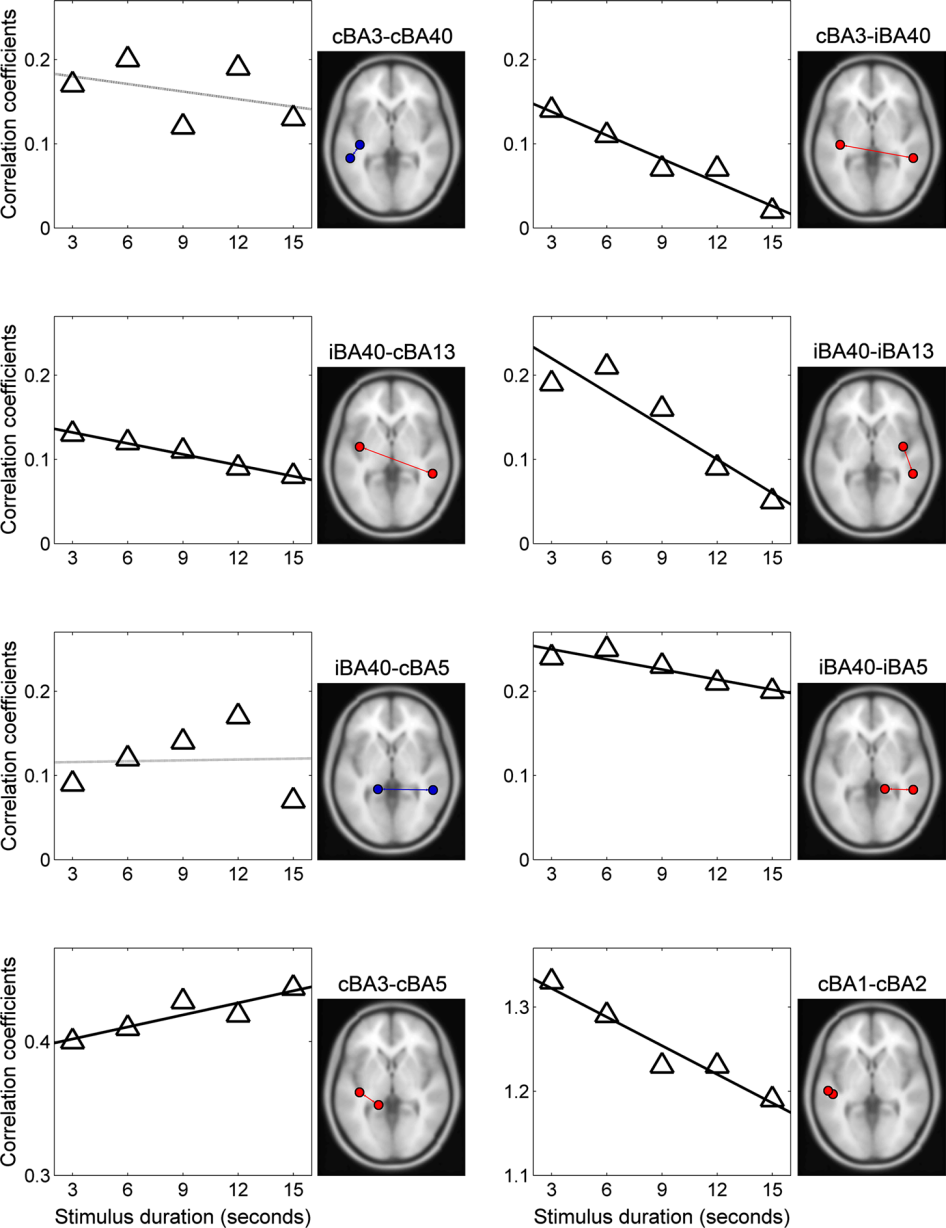
Linear decreases in inter-regional functional connectivity during pressure stimulation. The correlation coefficients (*y*-axis) in the inter-hemispheric (cBA3-iBA40 with *p* < 0.01 and iBA40-cBA13 with *p* < 0.001), ipsilateral (iBA40-iBA13 with *p* < 0.05 and iBA40-iBA5 with *p* < 0.05), and local cSI (cBA1-cBA2 with *p* < 0.01) connections significantly decreased during 15 s pressure stimulation. The correlation coefficients in one of the contralateral connections (cBA3-cBA5 with *p* < 0.05) significantly increased during 15 s pressure stimulation (linear regression analysis between the correlation coefficients and stimulus duration). Other thirty connections showed no significant change over time

The functional connectivity analysis revealed that Fisher-transformed correlation coefficients of the five connections, including cBA3-iBA40, cBA1-cBA2, iBA40-cBA13, iBA40-iBA13, and iBA40-iBA5, linearly decreased with time during 15 s pressure stimulation. Remarkably, it also revealed that the correlation coefficient in one connection of cBA3-cBA5 linearly increased with time (Fig. 4). The rates of decrease in the correlation coefficients were −0.008 *s*^−1^ in cBA3-iBA40 (*r*^2^ = 0.95, *p* < 0.01), −0.009 *s*^−1^ in cBA1-cBA2 (*r*^2^ = 0.94, *p* < 0.01), −0.003 *s*^−1^ in iBA40-cBA13 (*r*^2^ = 0.98, *p* < 0.001), −0.009 *s*^−1^ in iBA40-iBA13 (*r*^2^ = 0.87, *p* < 0.05), and −0.003 *s*^−1^ in iBA40-iBA5 (*r*^2^ = 0.84, *p* < 0.05). The rate of increase in the correlation coefficient was 0.003 *s*^−1^ in cBA3-cBA5 (*r*^2^ = 0.81, *p* < 0.05). The other thirty connections showed no significant change in the correlation coefficient over time.

## Discussion

### Tactile adaptation on the cortex

We made two observations in the cortical activation patterns during tactile adaptation: (1) that the number of activated regions reduced from the diverse somatosensory regions to the contralateral postcentral gyrus (SI) and (2) that the number of suprathreshold voxels of the contralateral SI decreased.

The first result may indicate that cortical regions are refined during adaptation for efficient sensory processing by gradually excluding cortical activities of the regions that are more involved in initial comprehensive perception of tactile stimuli but leaving only the primary somatosensory region active to sustain a basic function for tactile detection. The second result may be explained by the following two possibilities. First, activation of the contralateral SI becomes sharply tuned for stimulus detection by narrowing its spatial extent during adaptation, as illustrated in the sharpening model [39]. A previous optical intrinsic imaging study in monkeys reported that responses of forelimb regions in SI became spatially sharper but stronger by suppressing their surroundings during sustained vibrotactile stimulation for 5 s [4]. Although surrounding suppression was unobserved in our results, we conjecture its plausibility from a previous fMRI study showing deactivation of the ipsilateral SI and bilateral primary motor cortex during vibrotactile stimulation to the finger [40]. However, the sharpening model does not fully elucidate our results as we did not observe any temporal increase in activation levels (e.g., *F* or *Z* values) in the contralateral SI. Second, exponential decreases in the number of suprathreshold voxels of the contralateral SI may be associated with rapid information transfer from SI to other regions during adaptation [39]. Consequently, responses of the contralateral SI would be shortened, leading to a less number of activated voxels. This can also be supported by an increase in functional connectivity between the contralateral SI and PPC observed in the present study.

Our results showed exponential decreases in the number of suprathreshold voxels of the contralateral SI (cBA3, cBA1, and cBA2) and SII (cBA40) during 15 s pressure stimulation. These exponential decays in the contralateral SI and SII are similar to those in SA-I afferent adaptation [2] during sustained pressure stimulation. The response rate of the contralateral SII was much lower (*τ* of cBA40 = 20.99 s) than that of the contralateral SI. In terms of a hierarchical somatosensory network for tactile information processing from SI (low-level) to SII (high-level) [41], it is likely that these adaptive changes in the degrees of activation occurs in the contralateral SI earlier than in the contralateral SII. Other regions beyond the contralateral SII including the ipsilateral SII, bilateral PPC and insula showed no significant adaptive change in the degree of activation. It implies that cortical regions for earlier tactile processing [24] are apt to be foci during tactile adaptation.

### Functional connectivity during tactile adaptation

The functional connectivity analysis revealed three findings: (1) that inter-hemispheric (cBA3-iBA40 and iBA40-cBA13) and ipsilateral (iBA40-iBA13 and iBA40-iBA5) functional connectivity linearly decreased, (2) that contralateral (cBA3-cBA5) functional connectivity linearly increased, and (3) that contralateral (cBA1-cBA2) functional connectivity within SI linearly decreased.

The first finding is supported by previous fMRI studies in humans about a task-specific hemispheric dominance in tactile perception. It has been reported that the left hemisphere was dominantly involved in grating orientation [42], shape encoding [43], and the discrimination of vibrotactile frequency [36] while the right hemisphere was dominantly involved in grating location [42], shape matching [43], tactile pattern [36], and kinesthetic processing [44, 45]. As a simple static pressure was consistently applied to the same location for 15 s in our study, high-level tactile perceptual processes such as identifying tactile locations might become less active by adaptation-induced learning [39]. Hence, transmission of tactile information for stimulus location to/from the right (ipsilateral in our case) somatosensory cortex might decay over time. This is consistent with our observation of decreases in functional connectivity primarily with the ipsilateral SII: between the ipsilateral SII and contralateral SI, between the ipsilateral SII and bilateral insula, and between the ipsilateral SII and ipsilateral PPC [37, 46].

The second finding is associated with the roles of PPC in high-level tactile information processing [5, 24, 47, 48] and the anatomical evidence showing dense sensory projections from SI to BA5 [47, 48]. Note that the PPC alone did not exhibit any significant activation during tactile stimulation, but its connectivity to SI appeared to be enhanced during adaptation. From this, we speculate that increased connectivity between SI and PPC might rather reflect increased efficiency in somatosensory information transmission during adaptation than increases in high-level information processing of PPC. It may also indicate that information processing becomes more efficient during tactile adaptation, passing the information from cBA3 (the first area receiving sensory input) directly to cBA5 without much intermediate processes. This speculation is also supported by the third finding showing decreased connectivity between cBA3 and cBA2 within SI so that more efficient information transmission from cBA3 to cBA5 is made possible. Taken together, we postulate that the human somatosensory cortex adaptively adjusts both low- and high-level tactile perception processes during sustained pressure stimulation by changing the strength of inter-regional functional connectivity.

Adaptation of cortical activity in this study may be considered as an extension of the response profile at the afferent level in the peripheral nervous system, sharing a similar property such as exponentially decaying responses. However, we suspect that it may not be a mere reflection of afferent adaptation because of two distinct spatiotemporal properties: time constants (*τ*) of adaptation and changes in inter-regional interaction. First, we found that *τ* values at the contralateral SI were 5.69, 18.03, and 5.66 s at BA3, BA1, and BA2, respectively. While the average *τ* value over these SI sub-regions (9.79 s) was similar to the afferent time constant (8.40 s), individual sub-regions showed their own time constants which were apparently different from the afferent time constant. Second, changes in the firing activity of the peripheral afferents may not fully explain the adaptive changes in inter-regional functional connectivity at the cortical level. Region-specific variations (increase or decrease) in functional connectivity during sustained pressure stimulation can be considered as distinct adaptive behavior of somatosensory cortical activity. These spatiotemporal properties imply that adaptation of cortical activity may represent specific neural mechanisms dealing with inputs from adaptive sensory afferents. However, further studies are warranted to unveil precise relationships between afferent and cortical activity patterns during tactile adaptation.

### Limitations and future work

In line with previous non-fMRI neuroimaging and neurophysiologic studies on the cortical responses during tactile adaptation [49–54], we studied how cortical activity adaptively changed during pressure stimulation by investigating the degrees of cortical activation and inter-regional functional connectivity using fMRI. The following research topics, however, should be pursued in future work to fully corroborate our findings, which includes long-term adaptation, complexity of tactile stimulation (e.g., texture or shape), and correlations between cortical activation patterns and individual perceptual sensitivity during tactile adaptation.

## Conclusion

In the present study, we investigated changes in human cortical activity to sustained pressure stimuli using fMRI. During pressure stimulation lasting for 15 s, we found (1) that the number of suprathreshold voxels of the contralateral SI and SII exponentially decreased with time and (2) that inter- and intra-hemispheric inter-regional functional connectivity over the regions associated with tactile perception linearly decreased or increased with time. In particular, functional connectivity between the ipsilateral SII and other several regions decreased whereas functional connectivity between the contralateral BA 3 and 5 increased. In addition, functional connectivity between the contralateral BA 1 and 2 decreased. These findings suggest that cortical activation and inter-regional interactions adaptively changed during tactile adaptation for efficient tactile information processing.
